# More than Just a Simple Cardiac Envelope; Cellular Contributions of the Epicardium

**DOI:** 10.3389/fcell.2017.00044

**Published:** 2017-05-01

**Authors:** Angel Dueñas, Amelia E. Aranega, Diego Franco

**Affiliations:** Cardiac and Skeletal Muscle Research Group, Department of Experimental Biology, University of JaénJaén, Spain

**Keywords:** epicardium, proepicardium, non-coding RNAs, heart development, regeneration

## Abstract

The adult pumping heart is formed by distinct tissue layers. From inside to outside, the heart is composed by an internal endothelial layer, dubbed the endocardium, a thick myocardial component which supports the pumping capacity of the heart and exteriorly covered by a thin mesothelial layer named the epicardium. Cardiac insults such as coronary artery obstruction lead to ischemia and thus to an irreversible damage of the myocardial layer, provoking in many cases heart failure and death. Thus, searching for new pathways to regenerate the myocardium is an urgent biomedical need. Interestingly, the capacity of heart regeneration is present in other species, ranging from fishes to neonatal mammals. In this context, several lines of evidences demonstrated a key regulatory role for the epicardial layer. In this manuscript, we provide a state-of-the-art review on the developmental process leading to the formation of the epicardium, the distinct pathways controlling epicardial precursor cell specification and determination and current evidences on the regenerative potential of the epicardium to heal the injured heart.

The development of the heart is a complex process. The primitive heart tube is formed from cardiogenic mesoderm of the cardiac crescents, i.e., first heart field (FHF), while anterior and venous poles are derived from a subsequent subset of cardiogenic cells located medial to the cardiac crescents, dubbed second heart field (SHF; Kelly et al., 2001; Kelly and Buckingham, 2002). In addition, external cellular contributions to the developing heart will take place from this stage onwards. On the one hand, cardiac neural crest will colonize the most anterior parts of the heart playing a pivotal role on aortico-pulmonary septation (Kirby and Waldo, 1990, 1995). On the other hand, cell originating from the proepicardium (PE) will cover and infiltrate into the developing heart leading to distinct cellular subpopulations, such as endothelial and smooth muscle cells forming the coronary vasculature, endocardial cushion mesenchyme, cardiac fibroblasts, and of course the adult epicardial lining (Winter and Gittenberger-de Groot, 2007; Gittenberger-de Groot et al., 2012). In this manuscript we will provide a state-of-the-art review on the developmental process leading to the formation of the PE/epicardium, the signaling pathways providing cell specification and fate determination to those epicardial precursor cells including the upcoming role of non-coding RNAs, and current evidences on the regenerative role of the epicardium as to heal the injured heart.

## Initial phases of the proepicardial (PE) and epicardial formation; a journey to the developing embryonic heart

The proepicardium (PE) is a small protuberance that progressively develops within limiting boundaries between the hepatic and cardiac primordia. It is composed of an external epithelial lining configured as a cauliflower structure and an internal mesenchymal component (Virágh et al., 1993; Kálmán et al., 1995; Ratajska et al., 2008). A single PE anlage is observed at early developmental stages in zebrafish (Serluca, 2008) while in the sturgeon and in mice bilateral PE buds are formed subsequently merging into a single midline structure (Schulte et al., 2007; Icardo et al., 2009). Curiously, in chicken two PE primordia are formed, but interestingly the right PE anlage develops before the left one is visible (Schulte et al., 2007). These data suggest divergent evolutionary trends on the formation of the PE primordia and furthermore advocate that embryonic left-right signaling might play a role controlling PE formation (Schlueter and Brand, 2012).

**Figure 1:**
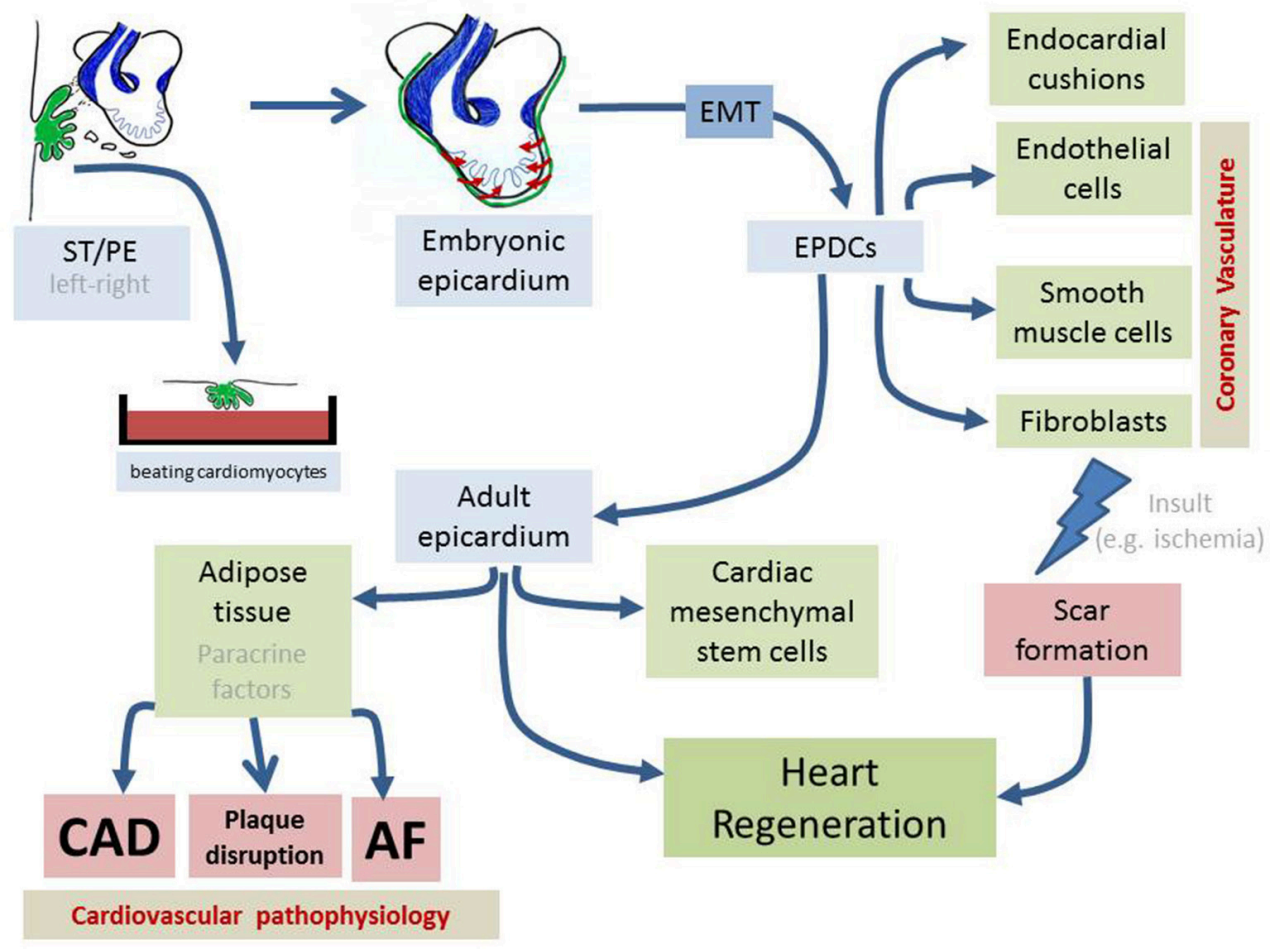
**Schematic representation of the distinct developmental stages of the proepicardium (PE)/septum transversum (ST) formation as well as on the distinct lineage contribution of the embryonic and adult epicardium**.

Transcriptional heterogeneity is widely documented for the PE anlage, and in addition, cell specific markers for several of the PE/epicardial cell derivatives, such as endothelial (Poelmann et al., 1993; Mikawa and Gourdie, 1996; Cossette and Misra, 2011; Niderla-Bielińska et al., 2015) and smooth muscle (Valder and Olson, 1994) cells have also been documented, suggesting an early heterogeneous compartmentalization. Subsequently after the formation of the PE a process of delamination and migration of the proepicardial cells occurs. This process will lead to external covering of the atrioventricular canal and the entire atrial and ventricular myocardial chambers as demonstrated by seminal studies using quail-chicken embryos (Pérez-Pomares et al., 1998, 2002; Vrancken Peeters et al., 1999; Figure 1). In zebrafish, this process is dependent on the pericardial fluid currents (Peralta et al., 2013, 2014; Plavicki et al., 2013, 2014). In mice, proepicardial cells are detached from the PE forming cysts that migrate to the developing cardiac chambers through the pericardial cavity (Männer et al., 2001; Hirose et al., 2006). These cysts randomly attach to the ventricular and atrial chambers and progressively expand until the final full coverage of the cardiac chambers is completed.

Once the PE cells migrate and cover the surface of the developing embryonic myocardium an epicardial-myocardial signaling crosstalk is initiated. This process is crucial for the correct development of both cardiac tissue layers. The epicardium is instructed to initiate an epithelial-to-mesenchymal transformation (EMT), detaching from the epithelial epicardial layer and migrating first into the subepicardial space. These cells subsequently invade the myocardial walls, giving rise to the epicardial derived cells (EPDCs) (Dettman et al., 1998). An additional source of subepicardial cells of hematopoietic origin is provided during embryonic development which further contributes to the heterogeneity of the embryonic and postnatal epicardium (Balmer et al., 2014). In the following chapters we provide a state-of-the-art review on the differential contribution of the embryonic epicardium in cardiovascular development and disease.

## Cell fate and contribution of the embryonic epicardium to the mature heart

Epicardial derived cells once they go through the subepicardial space continue their journey into the developing heart. Seminal approaches using quail-chick chimeras demonstrated that quail EPDCs contribute to distinct cardiac cell lineages, such as endothelial and smooth muscle cells in the coronary vasculature, endocardial mesenchymal cells in the atrioventricular cushions and also cardiac fibroblasts (Poelmann et al., 1993; Dettman et al., 1998; Figure 1). Since the experimental model used was a heterologous chimera, multiple criticisms were arising as which was indeed the real contribution of these cells. Supporting evidences were generated using retroviral-defective cell lineage tracing experiments in chicken hearts providing similar results (Mikawa and Gourdie, 1996); i.e., vascular endothelial, smooth muscle, and cardiac fibroblasts. Contribution to endocardial cushions is scarce, although it has been proposed that these cells are important for the correct development of the atrioventricular junction and the annulus fibrosus (Lie-Venema et al., 2008; Zhou et al., 2010; Lockhart et al., 2014). More recently, a contribution to cardiac resident stem cells (mesenchymal-like) has also been reported (Chong et al., 2011). In all cases, contribution to the developing myocardium was never observed (Poelmann et al., 1993; Mikawa and Gourdie, 1996; Pérez-Pomares et al., 2002). Surprisingly, *in vitro* PE culture experiments demonstrated that cardiomyocytes could be derived from these precursor cell pools (Kruithof et al., 2006).

With the advent of the molecular era, genetic lineage tracing in mice assaulted the quest to understand the contribution of the PE/embryonic epicardium to the mature murine heart. Several lineage tracing approaches were documented, in most cases, using Cre/loxP conditional activation of the reporter genes. In this setting, *Tbx18-*lineage tracing demonstrated a contribution to all the previously reported EPDC-derived lineages but surprisingly, also to the cardiomyocyte lineage. Whereas, these studies claimed that epicardial Tbx18+ cells contributed *in vivo* to ventricular cardiomyocytes (Cai et al., 2008), it was previously reported that fetal cardiomyocytes also expressed *Tbx18* (Franco et al., 2006; Christoffels et al., 2009; Zeng et al., 2011) and thus those Tbx18+ epicardial lineage tracing experiments were dubious.

Epicardial Wt1+ derived cells have also been reported to contribute to endothelial cells and to the myocardium (Zhou et al., 2008; Zhou and Pu, 2011). Evidence for Wt1+ cells in the embryonic heart has also been reported but excluding cardiomyocytes (Zeng et al., 2011) yet more recent evidence demonstrated that *Wt1-*derived cardiomyoctes can be traced in the developing heart before PE/epicardial formation (Rudat and Kispert, 2012; Cano et al., 2016), thus questioning the epicardial contribution to the developing cardiac muscle. On the other hand, prove that epicardial cells do not contribute to myocardium in zebrafish comes from *Tcf21*-tracing (Kikuchi et al., 2011) and transplant experiments (González-Rosa et al., 2012), in which a contribution to the perivascular beds is reported. While these data might support the notion that epicardial cells can contribute to the formation of cardiomyocytes *in vivo*, yet these evidences remain controversial, mainly because the limitation on the use of Cre-based techniques as a *bone fide* fate mapping approach (Christoffels et al., 2009).

Additional controversies have also arisen regarding the contribution of EPDCs to other vascular components. To date, it seems clear that EPDCs mostly contribute to cardiac fibroblasts and vascular smooth muscle cells, but their contribution to vascular endothelial cells have also been challenged by additional Cre-based fate mapping experiments. In fact, epicardial-derived Cre based lineage tracing in mice failed to provide substantial contribution to the developing vascular endothelium in mice (Merki et al., 2005; Cai et al., 2008; Zhou et al., 2008). Red-Horse et al. (2010) described that coronary endothelial lining was mostly entirely derived from the sinus venosus endothelium as revealed by an *Apelin-Cre* mice (Red-Horse et al., 2010; Tian et al., 2013), a process that is VEGF-dependent (Chen et al., 2014). However, additional evidences reported that ventricular endocardial cells also can contribute to the coronary vasculature (Wu et al., 2012) as revealed by *Nfatc1-Cre* lineage tracing. Furthermore, by the usage of novel proepicardial lineage tracing markers such as *Scleraxis-Cre, Semaphorin-3D-Cre*, and *Fabp4-CreER* drivers (Katz et al., 2012; He et al., 2014) a contribution to the coronary vasculature was also reported. In fact, reconciling evidences reported by Chen et al. (2014) determined that sinus-venous (SV) derived coronary vasculature mostly contributed to the dorsal and lateral coronary vasculature (~70%) whereas the ventral aspects were mostly endocardial derived (~70–80%), with just a small (~20%) but uniform contribution from the epicardium. These data are in line with a recent report that similarly estimated a 20% contribution from the proepicardium (Cano et al., 2016). Interestingly, a significant proportion of SV-derived and endocardial-derived cells displayed overlapping patterns with PE-derived cells, suggesting a common lineage origin. These data support the notion that multiple precursor cell populations contribute to the formation of the cardiac vasculature in mice, in contrast to avian hearts, in which the epicardial-derived contribution is large and undisputed. Lineage relationships between these three distinct coronary vasculature components remain nonetheless to be fully elucidated in mice.

Over the last decade our understanding of the molecular regulation of epicardial derived cells has largely increased with the usage of conditional spatio-temporal deletion of discrete signaling pathways. Epicardial cells display distinct divergent and overlapping expression patterns of *Wt1, Nfatc1, Tbx18*, and *Pod1* in the chicken and murine hearts (Braitsch et al., 2012), providing a heterogeneous panel of potentially distinct cardiac stem cells. Whereas, to date it remains elusive when and how epicardial cells becomes specific to their prospective lineage, it is increasing clear that multiple factors play pivotal roles in this process as summarized in Table 1. In particular, PDGFRβ is important for epicardial migration and for the development of coronary vascular smooth muscle cells (Mellgren et al., 2008; Bax et al., 2009; Smith et al., 2011), retinoic acid and VEGF primes endothelial vs. smooth muscle differentiation (Guadix et al., 2006; Tomanek et al., 2006; Azambuja et al., 2010) and Fgf signaling (Guadix et al., 2006), mainly through Fgf10 and Fgfr2b are essential for cardiac fibroblast formation (Vega-Hernández et al., 2011). In addition, *Pod1/Tcf21* is regulated by retinoic acid and inhibits differentiation of EPDCs into smooth muscle cells in chicken and mice (Braitsch et al., 2012) while Wnt signaling is also important for epicardial specification, as *Dkk1* and *Dkk2* mouse mutants display impaired epicardial development (Phillips et al., 2011). Similarly PCP disruption is also critical in this context (Phillips et al., 2008) as well as MAPK kinase genetic inactivation (Liberatore and Yutzey, 2004; Craig et al., 2010a,b). Other signaling pathways, such as *CXCL12/CXCR4* are also crucial for cardiac vascular development (Cavallero et al., 2015). Furthermore, Hippo signaling, mediated by *Yap/Taz* modulates *Tbx18* and *Wt1* expression in the epicardium controlling their contribution to the coronary vasculature (Singh et al., 2016). Several other molecules have also been reported to be critical for coronary artery formation, such as tenascin C (Ando et al., 2011) and nephrin (Wagner et al., 2011) particularly for smooth muscle recruitment to those cardiac vessels. Overall these findings highlight the complexity of distinct signaling pathways and molecules governing the coronary vasculature development.

**Table 1:** **List of transcription factors, growth factors are other distinct molecules involved in distinct phases of proepicardium/epicardium development**.

	**PE formation**	**EMT**	**Cell differentiation**	**References**
**TRANSCRIPTION FACTORS**				
wt1	Specification	Cell migration	Endothelial and myocardial cells	Zhou et al., 2008; Zhou and Pu, 2011; Rudat and Kispert, 2012; Cano et al., 2016
tbx5	Specification			Liu and Stainier, 2010; Diman et al., 2014
tbx18		Cell migration		Takeichi et al., 2013; Wu et al., 2013
tcf21/pod1			Inhibits SM cells; promotes fibroblasts	Braitsch et al., 2012
nkx2.5	Specification			Zhou et al., 2008
islet-1	Specification		Fibroblasts formation	Zhou et al., 2008; Brønnum et al., 2013a
gata-4	Specification			Watt et al., 2004; Kolander et al., 2014
Coup-tfII		Cell migration		Lin et al., 2012
Mrtf1/Mrtf2		Cell migration		Trembley et al., 2015
Nf1		Cell migration		Baek and Tallquist, 2012
**GROWTH FACTORS**				
Tgfb1	Tgf b signaling	Cell migration		Craig et al., 2010a
Tgfb2	Tgf b signaling	Cell migration		Craig et al., 2010a
Tgfbr3	Tgf b signaling	Cell migration		Sánchez and Barnett, 2012
fgf10	Fgf signaling		Fibroblasts	Guadix et al., 2006; Vega-Hernández et al., 2011
fgfr2b	Fgf signaling		Fibroblasts	Guadix et al., 2006; Vega-Hernández et al., 2011
dkk1	Wnt signaling			Phillips et al., 2011
dkk2	Wnt signaling			Phillips et al., 2011
cxcl12			Coronary vasculature contribution	Cavallero et al., 2015
ccr4			Coronary vasculature contribution	Cavallero et al., 2015
yap	Hippo signaling		Coronary vasculature contribution	Singh et al., 2016
taz	Hippo signaling		Coronary vasculature contribution	Singh et al., 2016
pdgfrbeta	PDGF signaling	Cell migration	SM cells	Mellgren et al., 2008; Bax et al., 2009; Smith et al., 2011
vegf		Cell migration	Endothelial cells	Guadix et al., 2006; Tomanek et al., 2006; Azambuja et al., 2010
**OTHERS**				
ra			Endothelial cells	Guadix et al., 2006; Tomanek et al., 2006; Azambuja et al., 2010
MEKK1	MAPK signaling	Cell migration		Craig et al., 2010b
tenascin c			SM cell recruitment	Ando et al., 2011
nephrin			SM cell recruitment	Wagner et al., 2011
Par6/Smurf/RhoA	Wnt signaling	Cell migration		Sánchez and Barnett, 2012
Vcam/RhoA		Cell migration		Dokic and Dettman, 2006

## The role of the postnatal epicardium in the injured heart

Within the adult heart, the epicardium represents the outermost layer, which is a simple epithelial layer. For many years, the functional role of this layer has been neglected as it was considered as an external cover devoid of any functional meaning. The discovery that the epicardial precursors can differentiate to beating cardiomyocytes has branded the epicardium as a source of cardiac stem cells with great therapeutic potential (Wessels and Pérez-Pomares, 2004; Pérez-Pomares et al., 2006; Winter et al., 2009). In addition, it has been reported that the adult epicardium plays a pivotal role in cardiac regeneration (Bollini et al., 2011, 2015; Schlueter and Brand, 2012; Masters and Riley, 2014; Kennedy-Lydon and Rosenthal, 2015; Figure 1) as detailed below.

Seminal work by Kruithof et al. (2006) described that the embryonic chicken PE if placed in appropriate cell culture conditions, was capable of giving rise to beating cardiomyocytes. Such *in vitro* conditions could be further promoted by Bmp administration and blocked by Fgf signaling. Thus, these data opened out the possibility that the epicardium could serve as an *in vivo* source of potential cardiomyocytes if the appropriate signals would be instructed *in vivo*. Importantly, Smart et al. (2011) demonstrated that adult epicardial derived cells, if previously primed with thymosinβ4, eventually generated functionally beating cardiomyocytes in an ischemic heart, yet the proportion of *de novo* integrated cells was rather spurious and its instructive mechanism remains rather obscure (Gajzer et al., 2013). Nonetheless, as a proof of principle approach it was highly valuable. This work introduced a novel concept of an activated epicardium, a condition by which embryonic epicardial markers such as *Wt1* and *Tbx18* are re-expressed in the adult epicardium (Huang et al., 2012; van Wijk et al., 2012; Braitsch et al., 2013; Bollini et al., 2014; Aguiar and Brunt, 2015) in response to distinct biological stimuli such as thymosinβ4 (Smart et al., 2012; Smart and Riley, 2012), stem cell factor (SCF; Xiang et al., 2014), and prokineticins (Urayama et al., 2008) among others. In addition, this activated epicardium secretes paracrine factors that modulate myocardial injury response (Zhou et al., 2011; Foglio et al., 2015).

While it is documented that the human heart has a limited capacity to regenerate (Bergmann et al., 2009), it is also highly acknowledged that the newt heart can also widely regenerate by other means (Becker et al., 1974; Oberpriller and Oberpriller, 1974). Furthermore, the adult zebrafish heart can also regenerate (Poss et al., 2002) and the epicardium provides a pivotal role during this regeneration process (Gemberling et al., 2015; Wang et al., 2015). Molecular analyses have demonstrated that the epicardium becomes activated as soon as the heart is injured and such activation provides instructive signals that promote cardiomyocyte proliferation, revascularization, and tissue repair (Lien et al., 2006, 2012; Marín-Juez et al., 2016). During this process a transitory scar stage occurs and is subsequently replaced by fully functional and integrated cardiomyocytes (González-Rosa et al., 2011; Mercer et al., 2013; Itou et al., 2014; Marro et al., 2016).

Further analyses in this front identified that Wnt1/β-catenin is crucial promoting formation of cardiac fibroblasts and hence cardiac repair (Duan et al., 2012). Several studies have identified key molecules modulating this regeneration capacity. For example, Nrg1 acts as a mitogenic agent in cardiomyocytes following injury during cardiac zebrafish regeneration (Gemberling et al., 2015). Notch (Zhao et al., 2014), Raldh2 (Itou et al., 2014), and myocardial NF-κB (Karra et al., 2015) are also essential for heart regeneration in zebrafish. Hydrogen peroxide (Han et al., 2014) has been reported to prime heart regeneration and telomerase has been identified as instrumental for zebrafish regeneration (Bednarek et al., 2015), but still it remains to be established if these factors are modulated by the epicardium. More recently, it has been demonstrated that epicardial regeneration is guided by the cardiac outflow tract and hedgehog signaling (Wang et al., 2015) and single cell transcriptome of the epicardium has identified caveolin1 as an essential factor in regenerating zebrafish heart (Cao and Poss, 2016). Moreover, re-expression of epicardial developmental genes and enhanced EMT in response to injury has been widely demonstrated (Lepilina et al., 2006; Kim et al., 2010; González-Rosa et al., 2011; Schnabel et al., 2011). These data suggest that complex regulatory networks control zebrafish regeneration (Rodius et al., 2016) positioning the epicardium as a key tissue layer for regeneration. Thus, these data will be highly instrumental to search for novel ways to heal the injured heart.

In adult mice, the regenerative capacity is lost and the injured heart responds by generating a fibrous scar which is derived from pre-existing epicardial cells (Zhou et al., 2008; Duan et al., 2012) as well as *de novo* recruited bone marrow-borne circulating cells (Ruiz-Villalba et al., 2015). Interestingly, full regeneration is achieved at early developmental stages, i.e., on the first week of life, in which the epicardium (Porrello et al., 2011) is also a highly instructive player and thymosinβ4 priming increases the time window for mammalian heart regeneration (Rui et al., 2014). In addition a role for Wnt signaling has also been identified in the regenerating heart in mice (Mizutani et al., 2016). Recent evidence demonstrated that exosomal signaling from the epicardium is essential for myocardial maturation highlighting a pivotal role for clustering in this process (Foglio et al., 2015). All these efforts have provided the bases of heart regeneration. A giant step was recently reported by Wei et al. (2015) whom used reconstitution of epicardial follistatin-like1 expression in biomaterial patches to heal the adult injured heart, opening a novel way to regenerate the adult mammalian heart.

## An unexpected epicardial derivative with paracrine signaling leading to CAD and AF

While it is highly acknowledged that the epicardial precursor cells, within the PE, and subsequently the EPDCs will give rise to distinct cardiovascular embryonic cell lineages, it has remained unexplored if the adult epicardium can generate additional cellular subpopulations. Recent evidences have demonstrated that intramyocardial adipose tissue is derived from the endocardium (Zhang et al., 2016), whereas adipose tissue around the heart, mainly at the venous, arterial connections, and atrial appendages is an adult epicardium derivative (Yamaguchi et al., 2015). Furthermore, cardiac adipose tissue deposition has recently been associated to distinct cardiovascular pathologies (Figure 1), such as coronary arteries diseases (Iwayama et al., 2014), atherosclerosis plaque disruption (Talman et al., 2014; Yamashita et al., 2014), and atrial fibrillation (Batal et al., 2010; Nakanishi et al., 2012; Gaborit et al., 2013). Although, these are early days to fully understand the molecular mechanisms linking epicardium, adipose tissue deposition, and cardiovascular pathologies, supporting evidences suggest that these cells can act as paracrine signaling center that, if impaired, can be the source of cardiovascular diseases (Langlois et al., 2010; Greulich et al., 2012).

## Non-coding RNAs in the PE/epicardium

Over the last decade we have witnessed a revolution in the concept of the control of gene expression with the discovery of non-coding RNAs. Non-coding RNAs can be broadly classified according to the transcript size into long non-coding RNAs (lncRNAs) and small non-coding RNAs. Our current understanding of lncRNAs is still in its infancy with just a limited number of reports in the developing heart (Grote et al., 2013; Klattenhoff et al., 2013; Sauvageau et al., 2013; Zhu et al., 2014; Kurian et al., 2015). On the other hand, our knowledge on the functional role of small non-coding RNAs, in particular microRNAs, has been largely increased (Callis and Wang, 2008; Chen and Wang, 2012; Bonet et al., 2013; Philippen et al., 2015; Yan and Jiao, 2016). microRNAs are small non-coding RNA of 18–24 nt in length that by homologous base-priming are capable of blocking translation or degrading mRNA transcripts. microRNAs are transcribed by RNA polymerase II, 5′ capped and 3′ polyadenylated leading to mature microRNA by RNA endonucleases such as Drosha and Dicer (Aranega and Franco, 2015; Towler et al., 2015). Mature microRNAs are loaded into the RISC complex which can thereafter search for mRNA transcript base complementarity (Hammond, 2015; Shen and Hung, 2015). To date more than a 1,000 distinct microRNAs have been identified in humans, which are quite conserved among evolution. A seminal study by Singh et al. (2011) demonstrated that conditional ablation of Dicer, an RNAse processing enzyme, in the epicardium provoked impaired epicardial formation, thin-walled myocardium, and aberrant coronary vasculature formation. Thus, this study demonstrated a pivotal role for microRNAs in PE/epicardium development. A large array of studies have been reported in key developmental processes by which the PE/epicardium is formed, such as epithelial-to-mesenchymal transition in cancer (see for recent reviews; Behbahani et al., 2016; Peng et al., 2016; Sulaiman et al., 2016; Zou et al., 2016) and also within the heart (Stankunas et al., 2010; Bonet et al., 2015) and cardiac regeneration (Porrello et al., 2013) but surprisingly only a short list of studies have been reported in PE/epicardium formation. miR-21 has been reported in numerous studies promoting fibrogenesis both during cardiac development and disease (Thum et al., 2008; Adam et al., 2012; Derda et al., 2015; Gupta et al., 2016). Brønnum et al. (2013a) has recently reported that miR-21 promotes fibrogenic EMT in epicardial cells by modulating *Pcd4* and *Sprouty-1* and these authors (Brønnum et al., 2013b) have also reported that islet-1 can influence miR-21 expression and therefore modulate cardiac fibrogenic EMT. Seeger et al. (2016) demonstrated that *let-7* inhibition enhances the recruitment of epicardial cells after myocardial infarction promoting an improved cardiac function. Overall these studies demonstrate a nascent role for microRNAs in PE/epicardium formation, which might provide novel approaches to activate and prime epicardial cells for cardiac regeneration.

## Conclusions and perspectives

Over the last decade our understanding of the cellular contribution of the PE/epicardium has largely increased. Seminal works using quail-chick chimeras demonstrated a large plasticity for the EPCDs, contributing to the cardiac fibrous skeleton, the coronary vasculature and the developing atrioventricular valves (Poelmann et al., 1993; Wessels and Pérez-Pomares, 2004; Figure 1). However, with the advent of molecular tracing tools, multiple evidences demonstrated a rather more complex contribution and architecture to the coronary vasculature in mice. Cre-driven fate mapping can be pervasive and promiscuous tools, deriving in complex and in many cases controversial findings. We hope that either retrospective clonal analysis as previously reported for myocardial components (Meilhac et al., 2003, 2004a,b) or genuine prospective lineage tracing would serve to reconcile these findings in the PE/epicardial context. With no doubt one of the seminal work that prompted the interest of the epicardial lining in the context of cardiac stem cell and cardiac regeneration was reported by Kruithof et al. (2006) demonstrating that PE/epicardial cells could be generating cardiomyocyte *in vitro* opening the possibilities to unlock the myocardial lineage commitment *in vivo*. Thymosin beta4 was the first of these unlocking tools, providing an entry site to regenerate the heart using the epicardium as a cell source (Smart et al., 2011; Smart and Riley, 2012). In addition, bridging epicardial activation by follistatin-like1 into biomaterials provided additional convincing evidences on the feasibility of these approaches (Wei et al., 2015). New tools will be discovered in the near future.

In recent years a novel link between the epicardium and epicardial derived structures is emerging (Figure 1). Intriguingly, adipose fat deposition within the pericardiac regions has been linked to cardiac pathophysiologies such as coronary artery atherosclerosis and atrial fibrillation. To date the casual relationship remains enigmatic, yet a plausible embryonic link might be present since epicardial cells can differentiate into adipose tissue (Zhang et al., 2016) and epicardial cells contribute to both endothelial and smooth muscle components of the coronary vessels (Pérez-Pomares et al., 2002; Cano et al., 2016). However, our current understanding is still in its infancy and for sure we will witness additional cellular and molecular evidences deciphering the interplay between these rather apparent distinct cardiovascular entities.

While our cellular and molecular understanding of PE/epicardium/EPDC has greatly advanced in recently years, the discovery of novel levels of gene regulations, in particular those exerted by the non-coding RNAs, is called to change our molecular and signaling pathways schemes. The discovery that microRNAs are crucial to epicardial development is simply demonstrating the equally pivotal roles of these tiny molecules in other cardiovascular developmental contexts (Cordes and Srivastava, 2009; Chinchilla et al., 2011; Bonet et al., 2015). In addition to microRNAs, long-non-coding RNAs are also called to play pivotal role in cardiogenesis (Grote et al., 2013; Klattenhoff et al., 2013) and thus similarly in epicardial development. In coming years, additional routes would be discovered demonstrating the essential role of these new players in epicardial biology both during development and disease.

## Author contributions

AD searched for literature records and wrote part of the manuscript. DF wrote part of the manuscript, editing, and approved the final manuscript version. AA read and suggested editing comments to the manuscript.

## Funding

DF and AA are partially funding by a grant of the Ministerio de Economia y Competitividad (BFU2015-67131-P) of the Spanish Government and the Consejeria de IEconomia y Conocimiento (CTS-446) of the Junta de Andalucia Regional Council.

### Conflict of interest statement

The authors declare that the research was conducted in the absence of any commercial or financial relationships that could be construed as a potential conflict of interest.

## Abbreviations

Bmp: Bone morphogenetic protein
CXCR4: Chemokine (C-X-C motif) receptor 4
Cre: Cre recombinase
CXCL12: Chemokine (C-X-C motif) ligand 12
Dkk1: Dickkopf-related protein 1
Dkk2: Dickkopf-related protein 2
EMT: Epitelial-to-mesenchymal transition
EPDCs: Epicardial derived cells
Fabp4: Fatty acid binding protein 4
Fgf: Fibroblast growth factor
Fgf10: Fibroblast growth factor 10
Fgfr2b: Fibroblast growth factor receptor 2b
FHF: First heart field
lcnRNA: Long non-coding RNA
MAPK: Mitogen-Activated Protein Kinase
Nfatc1: Nuclear factor of activated T-cells, cytoplasmic 1
NF-κB: Nuclear Factor κB
Nrg1: Neuregulin 1
Pcd4: Programmed Cell Death 4
PCP: Planar cell polarity
PE: Proepicardium
Pod1/Tcf21: Podocyte-expressed 1/Transcription factor 21
Raldh2: Aldehyde dehydrogenase family 1, subfamily A2
SHF: Second heart field
Tbx18: T-box homeobox 18
Tcf21/Pod1: Transcription factor 21/Podocyte-expressed 1
VEGF: Vascular endothelial growth factor
Wt1: Wilms tumor protein
Yap/Taz: Yes associated protein 1/Transcriptional coactivator with PDZ-binding motif.
